# A case of intestinal obstruction in a 90-year-old patient at a tertiary hospital in Central Saudi Arabia with a rare cause: obturator hernia!

**DOI:** 10.1093/jscr/rjac044

**Published:** 2022-02-21

**Authors:** Zeinah Sulaihim, Lina Alsaadon, Roaa Saleh Alsuhaibani, Hana Alfaleh, Ibrahim Albabtain

**Affiliations:** College of Medicine, King Saud bin Abdulaziz University for Health Sciences, Riyadh, Saudi Arabia; College of Medicine, King Saud bin Abdulaziz University for Health Sciences, Riyadh, Saudi Arabia; Department of General Surgery, King Abdulaziz Medical City, Ministry of National Guard, Riyadh, Saudi Arabia; Department of Radiology, King Abdulaziz Medical City (KAMC), Riyadh, Saudi Arabia; Department of Surgery, Ministry of the National Guard-Health Affairs, King Saud bin Abdulaziz University for Health Sciences, King Abdullah International Medical Research Center, Riyadh, Saudi Arabia

## Abstract

Obturator hernia is a pelvic floor type of hernia in which abdominal or pelvic contents protrude through the obturator foramen. It is considered rare in patients with signs and symptoms of intestinal obstruction causing a diagnostic challenge for clinicians. This case reports a 91-year-old multiparous female who presented with vague lower abdominal pain associated with obstipation and vomiting. We present a successful laparoscopic repair of obturator hernia in an elderly female.

## INTRODUCTION

Obturator hernia accounts for <1% of all types of hernias, proving its rareness and difficulty in diagnosis [1]. Due to its late diagnosis and treatment, almost half of the patients (47%) have high morbidity and mortality rates [2]. It is commonly found in elderly, multiparous female patients, with chronic illnesses and a low body mass index (BMI; [3]). This can be explained by women having a broader pelvis and larger obturator canals, which are usually 2–3-cm long and 1-cm wide [2, 3]. Patients usually present with nonspecific symptoms ranging from abdominal or groin pain to intestinal obstruction symptoms accompanied by nausea and vomiting [4]. Computerized tomography is a highly sensitive diagnostic method before surgery, where it allows proper abdominal and pelvic visualization of the hernia location, therefore, it helps surgeons to rely on the images for laparoscopic hernia repair rather than laparotomy [5, 6]. The aim of this study is to present a case of a patient with signs and symptoms of intestinal mechanical obstruction, which was diagnosed as an obturator hernia proven by computed tomography (CT) and treated by laparoscopic obturator hernia repair in King Abdulaziz Medical city.

## CASE REPORT

This case is for a 90-year-old female weighing 37.2 kg with a height of 155 cm (BMI = 15.48 kg/m^2^) who is a known case of HTN with a previous history of open cholecystectomy 30 years ago and femur fracture fixation. She presented to the emergency department with vomiting for an hour associated with diffuse lower abdominal pain, and obstipation for a long nonspecific time. The vomit was mainly food content without mucus or blood. The pain was associated with abdominal distention and loss of appetite. On examination, she was vitally stable, cachectic with a very small and tiny abdomen. The abdomen was distended, soft and lax with tenderness mainly in the lower abdomen. No signs of peritoneal irritation, and no hernia were appreciated. Howship-Romberg sign was not assessed upon examination as patient refused completion of examination.

**Figure 1:**
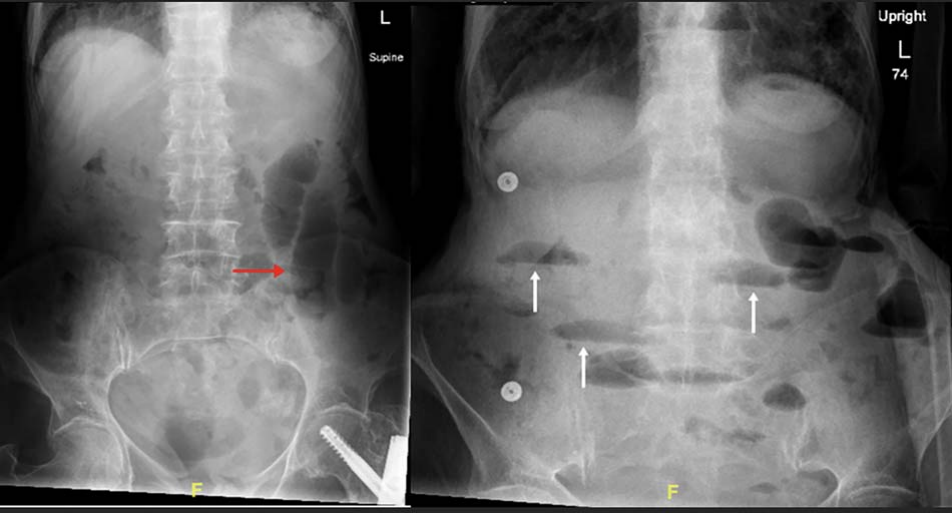
Supine and erect abdominal radiograph demonstrate distended small bowel loops (red arrow) with multiple air fluid levels (white arrow) suggesting small bowel obstruction.

**Figure 2:**
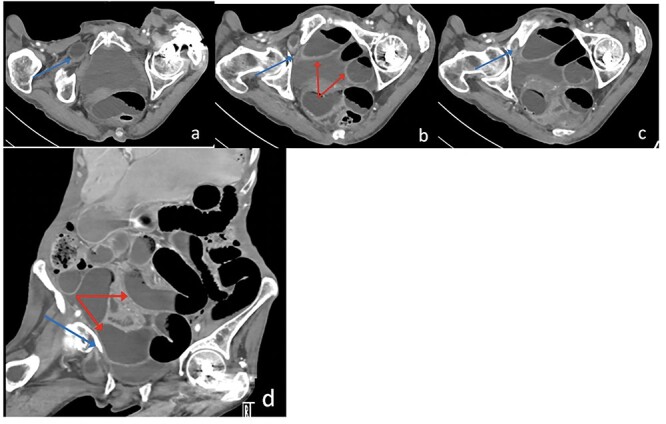
Axial (**a**–**c**) and Coronal (**d**) contrast-enhanced reformatted CT image of the abdomen shows distal ileal loop segment protrudes through the right obturator foramen with the herniated segment trapped between the right obturator externus and pectineus muscles (blue arrow), there is proximal upstream dilated small bowel loops (red arrows). In keeping with high-grade small bowel obstruction due to strangulated obturator hernia.

The patient’s lab results revealed leukocytosis of (12.40 m/mm^3^), and C-reactive protein of (28 mg/l). Abdominal X-ray demonstrated distended small bowel loops with multiple air fluid levels (Fig. 1). Moreover, CT revealed right sided obturator foramen hernia containing a segment of distal ileum causing high-grade small bowel obstruction, which reached up to 4 cm (Fig. 2). The CT also showed extensive bronchiectasis and consolidation with mucus plugging in the lung base.

**Figure 3:**
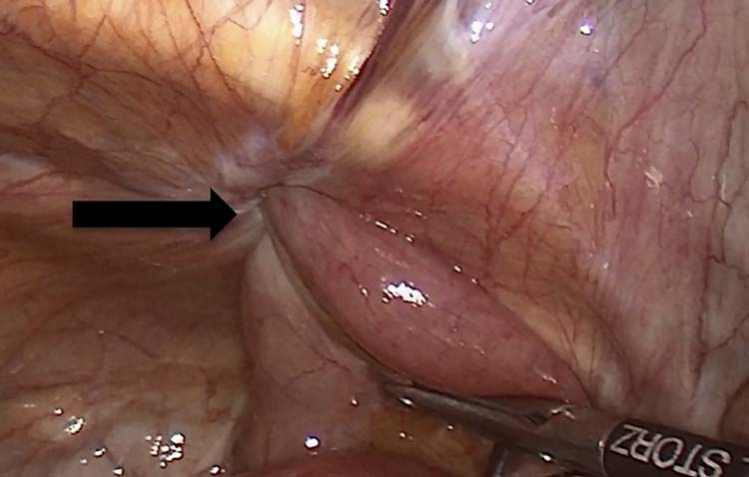
An intraoperative figure of the obturator hernia transitional zone revealing the hernial sac and protruded bowel segment.

**Figure 4:**
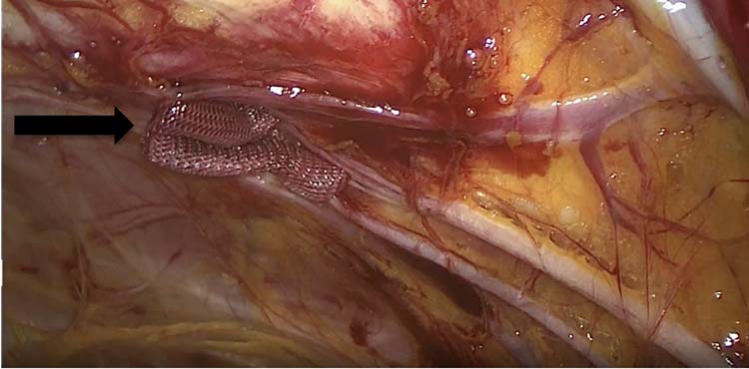
An intraoperative figure of the Vicryl mesh plug inserted into the obturator opening.

**Figure 5:**
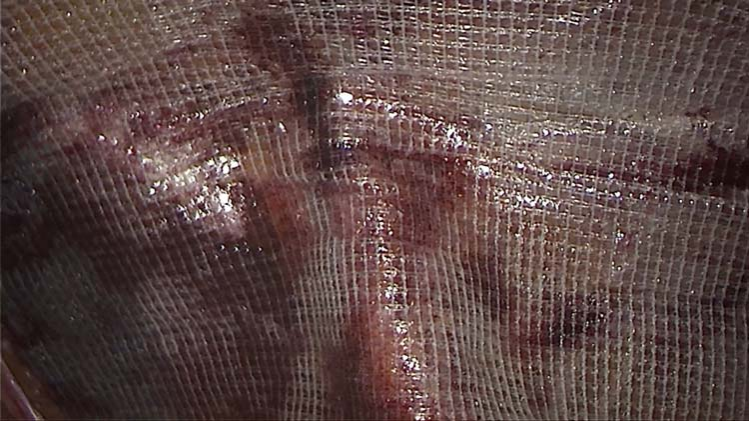
An intraoperative figure of the right ProGrip mesh covering the whole right area.

Due to the high-grade small bowel obstruction, the patient was pushed as an emergency case to the operating room. The surgery started and ended as a laparoscopic procedure with three small incisions; 12, 11 and 5 mm port sizes located supraumbilically, right and left midclavicular line relatively. Once the peritoneum was penetrated, and gas insufflated, diagnostic laparoscopy was done, and the obturator hernia with bowel content was visualized (Fig. 3), with a transitional zone at the hernia site showing a proximal dilatation and distal collapse of the small bowel. After that, reduction of the hernia content was subsequently made with no signs of gangrene or ischemia of the bowel. Then, the small bowel was run as a whole to eliminate any other transitional zones or pathology, which was unremarkable. Therefore, as there was no contamination, ischemia or perforation, a Vicryl mesh was elected and inserted as a plug into the obturator opening using a peritoneal flap and fixed (Fig. 4). Once the fixation was obtained, a ProGrip mesh was then applied to cover the whole right area (Fig. 5), which was covered by the peritoneum afterwards. The abdomen was inspected and the bowel looked healthy. Lastly, the ports were removed, the incisions were closed, the skin was clipped and then the dressing was applied.

Postoperatively, the patient was admitted to the intensive care unit for 1 day due to the high risk of surgery and to be observed carefully by a multidisciplinary team including the surgical team. Then, she was shifted in good condition to the surgical ward for 2 days. During her admission, the patient was vitally stable, conscious, communicating, not in pain and tolerating oral liquid diet. Moreover, the dressing was changed daily and revealed no signs of surgical site infection. On her second postoperative day she was able to ambulate, however was not discharged until the next day after she had a bowel motion. Two weeks later, she was followed by the consultant in the outpatient clinic for clip removal. The surgery and all health care workers involved in taking care of the patient proved the success of the treatment plan as the patient was sent home in good health without any complications.

## DISCUSSION

The obturator hernia is defined as a rare type of protrusion of abdominal content into the obturator canal, which is bounded by the obturator muscle medially and the pectineus muscle laterally [4]. It is commonly found in elderly multiparous women with low BMI and chronic illnesses with obstructive lung diseases being the most common due to increase in the intra-abdominal pressure during coughing, similar to our patient who had bronchiectasis [1].

It is diagnostically challenging as it has nonspecific abdominal obstruction symptoms [3]. Moreover, a diagnosis of obturator hernia can be made preoperatively by CT as it has high sensitivity and specificity, however, most patients suffering from obturator hernias were diagnosed intraoperatively during diagnostic laparoscopy, which is different from our case [1, 6, 7]. The management of this type of hernia is surgical repair as it is the gold standard. In the literature, obturator hernia repair is done as an open procedure, however, in our case, the surgery was initiated and completed as a laparoscopic procedure due to the advantage of shorter length of hospital stay, less postoperative pain and lower risk of complications [1, 6, 8].

A hernia repair can be either with interrupted sutures or with mesh application. A retrospective analysis of 80 obturator hernia repairs was published in 2013 and found a 3-year recurrence rate of 22% for patients who have not had a mesh repair. On the other hand, 0% recurrence rate was found in the mesh repair group [9]. In this case, a synthetic ProGrip mesh was inserted and fixed and no recurrence was identified by the time of writing this case report.

## CONCLUSION

Obturator hernia is a challenging diagnosis and an important cause of bowel obstruction with high morbidity and mortality rate [2]. This report aims to remind health care professionals to include obturator hernia as a differential diagnosis for any elderly, thin and multiparous female with signs and symptoms of intestinal obstruction.

## CONFLICT OF INTEREST STATEMENT

None declared.

## FUNDING

None.
